# Dudley’s Gynecology

**Published:** 1900-06

**Authors:** 


DUDLEY'S GYNECOLOGY.—A treatise on the principles and practice of Gynecology. By E. C. Dudley, A. M., M. D., Professor of Gynecology in the Northwestern University Medical School, Chicago. New (2nd) edition. In one very handsome octavo volume of 717 pages, with 453 engravings, of which 47 are in colors and 8 colored plates. Just ready. Cloth, $5.00, net; leather, $6.00, net. Lea Brothers & Co., 710 Sansom Street, Philadelphia. 1900.

This book covers the entire subject from a practical standpoint.

It is not burdened with speculative and theoretical views, and its arrangement is primarily pathological and secondarily regional. This gives the practitioner, first, the disease, and, last, the treatment.

The first edition of this work was exhausted in a year, which shows its great popularity. A searching revision has been made in the new edition. Seventy-eight new pages have been added, besides thirty-one engravings and a number of full-page plates.

This is a strong book, and is deservedly popular. T. J. B.

